# Entropy signatures of interstate aggression on social cohesion dynamics

**DOI:** 10.1098/rsos.251310

**Published:** 2025-11-12

**Authors:** Jais Adam-Troian, Nathalie Bauchet, Arsalane Chouaib Guidoum, Abdessadek El Ahmadi

**Affiliations:** ^1^ School of Social Sciences, Heriot-Watt University Dubai, Dubai, UAE; ^2^ Aix-Marseille University, Marseille, France; ^3^ Department of Mathematics and Computer Science, University of Tamanghasset, Tamanrasset, Tamanrasset Province, Algeria

**Keywords:** entropy, warfare, aggression, Ukraine, themodynamics, social cohesion, dynamical systems

## Abstract

This study proposes a systems approach to explore the entropy signatures of war phases on social cohesion dynamics in Ukraine (2004–2025). Using a psycholinguistic time series of online searches for first-person plural pronouns, we quantified social cohesion complexity using sample entropy and refined composite multiscale entropy (RCMSE). Analyses reveal distinct signatures between war phases over time for both metrics, with, respectively, *R*^2^ = 0.57 and *R*^2^ = 0.95. Relative to peace and full invasion, social cohesion entropy decreased under partial invasion and displayed more anti-persistent behaviour. While social cohesion was most dysfunctional under partial invasion, entropy signatures of full-scale invasion and peace were relatively close. These results offer insights into the impact of interstate aggression on social cohesion and contribute to a dynamical systems understanding of warfare as a systemic shock. The methodology provides a framework for monitoring and predicting societal resilience in response to significant events.

## 1. Introduction

Social cohesion can be defined as the sense of belonging, connectedness and solidarity among individuals within a group [1]. Research spanning the Middle East, North Africa and Europe (including Ukraine) demonstrated that social cohesion is a universal predictor of will-to-fight among members of regular, irregular militaries, criminal gangs and the public [2]. In turn, analyses of modern warfare reveal that the will-to-fight (i.e. morale) is a stronger predictor of battlefield success than the ratio of combatant forces, on par with logistics and intelligence [3,4]. Therefore, the accurate description of social cohesion dynamics is key to understanding the will-to-fight of nations and their success in warfare.

To characterize social cohesion, we take a dynamical systems approach and examine how its behaviour is affected under peace and wartime. In line with prior empirical research, we assume that human coordination, synchrony and social behaviour follow the laws of thermodynamics [5]. This enables the conceptualization of national cohesion as a dynamical system which can transition between disordered states and more stable ones: from societal chaos (‘anomie’) to optimal functioning to dysfunctional hyper-rigidity (fascism). In this framework, warfare is a shock capable of triggering systemic changes ranging from mild perturbations to abrupt changes in system behaviour (i.e. catastrophes). We can therefore describe cohesion dynamics across different levels of shock to study its impact on the system.

An important systemic indicator is entropy, the degree of complexity in a given signal. Entropy levels of biological signals (e.g. gait, heart rates) is associated with the ‘healthiness’ of the underlying system: lower entropy is generally associated with pathological states such as ageing or diabetes [6]. Decreases in bio signal complexity are often interpreted as early signs of systemic dysfunction [7]. Here, we sought to quantify entropy levels of social cohesion over time and across levels of war. Owing to its geopolitical relevance, long range of available online data, and clearly identifiable warfare stages, we decided to focus on Ukraine, and to characterize its social cohesion entropy dynamics across phases of peace (pre-2014), partial annexation (low intensity warfare, 2014–2022) and full-scale invasion (high intensity warfare, post-2022).

## 2. Data and preliminary analysis

In this section, we analyse weekly Google Trends frequencies [8] for first-person plural pronouns in Ukrainian and Russian (2004–2025), used as a proxy for collective identity salience. From these series, we construct a normalized cohesion index capturing fluctuations in social cohesion. A Kruskal–Wallis permutation test [9] confirms significant differences across the three phases, providing the basis for the subsequent entropy-based analysis.

### 2.1. Raw data

**Figure 1.**
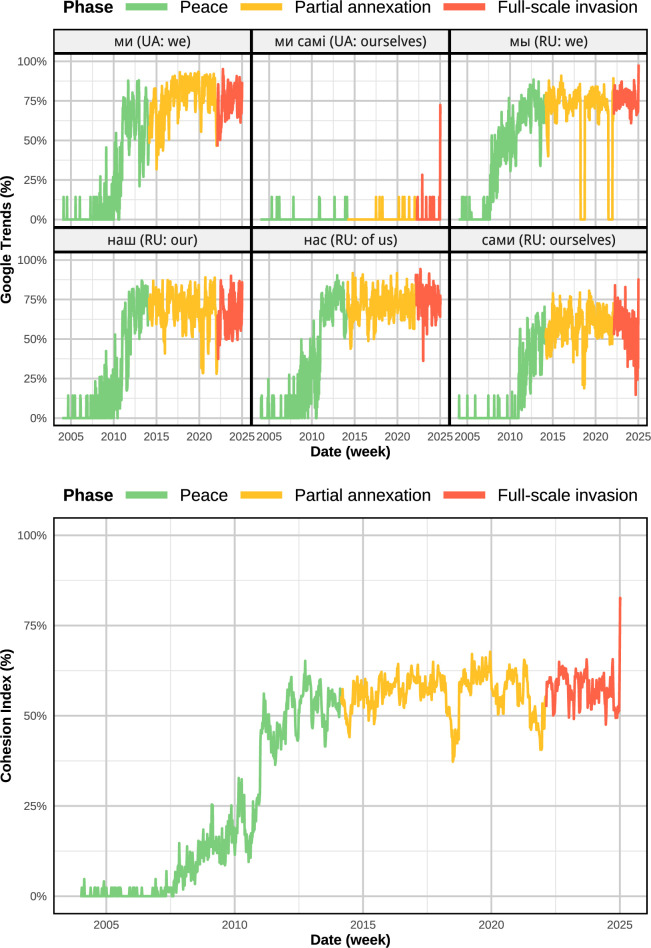
Psycholinguistic indicators of social cohesion in Ukraine. Top: weekly Google Trends frequencies for first-person plural pronouns. Bottom: cohesion index C(t) derived from the normalized average of the six pronoun series.

We rely on weekly Google Trends frequencies for first-person plural pronouns in both Ukrainian and Russian (‘нас’, ‘мы’, ‘наш’, ‘сами’, ‘ми’, ‘ми самi’). Using the method described in the electronic supplementary materials A.1 we extracted a social cohesion index from the psycholinguistic time series of online search terms for first-person plural pronouns in Ukraine (e.g. ‘we’, ‘us’, ‘ourselves’). This series is a validated measure of common ingroup identity and reliably predicts various offline collective behaviour [10]. We obtained a raw weekly time series spanning n=1099 weeks from 28 December 2003 to 12 January 2025. The top panel of figure 1 shows the unprocessed series, providing a first descriptive overview of cohesion-related search activity.

To obtain a synthetic measure of social cohesion, we define the following cohesion index:


(2.1) $$C(t)=\frac{1}{k}\sum_{j=1}^{k}x_{j}(t),$$

where $x_{j}(t)$ denotes the normalized frequency of term j in week t, and k is the number of terms included. The bottom panel of figure 1 illustrates the resulting cohesion index series C(t).

### 2.2. Permutation test for the cohesion index C(t)

To formally assess whether cohesion levels differed across phases ($F_{1}$: peace, $F_{2}$: partial annexation, $F_{3}$: full-scale invasion), we applied a permutation-based Kruskal–Wallis test. The hypotheses null/alternative can be stated as


(2.2) $$H_{0}:F_{1}=F_{2}=F_{3},\;H_{a}:\exists \;(i,j)\;\text{such that}\;F_{i}\neq F_{j},$$

where the Kruskal–Wallis statistic is defined as


(2.3) $$\chi_{\text{cal}}^{2}=\frac{12}{N(N+1)}\sum_{j=1}^{k}n_{j}{\left({\bar{R}}_{j}-\frac{N+1}{2}\right)}^{2},$$

with N the total sample size, $n_{j}$ the sample size in group j, and ${\bar{R}}_{j}$ the average rank in group j. In our application, the test statistic was $\chi_{\text{cal}}^{2}=605$, yielding a permutation p-value of less than $10^{-5}$. This result provides strong evidence against the null hypothesis $H_{0}$ and indicates that the distribution of the cohesion index, as defined in equation (2.1), differs significantly across the three phases.

**Figure 2.**
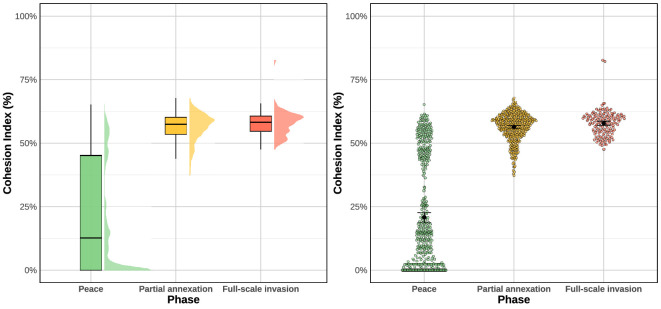
Cohesion index across phases. Left: a half-eye density plot with boxplots. Right: a beeswarm plot with individual observations, mean values and bootstrapped confidence intervals.

To provide a more detailed overview of the distribution of the cohesion index C(t) across the three phases, we present two complementary visualizations. Figure 2 displays, in the left-hand panel, a half-eye density plot combined with boxplots, thereby illustrating both the overall distributional shape and key summary statistics (median and quartiles). The right-hand panel shows the same data as a beeswarm plot, where individual observations are displayed together with mean values and bootstrapped confidence intervals. Taken together, these complementary perspectives highlight consistent differences across phases (peace, partial annexation and full-scale invasion), reinforcing the conclusions drawn from the statistical tests.

In summary, this section introduced the raw cohesion-related time series, defined a synthetic cohesion index, and provided initial statistical evidence of significant differences across phases. Subsequently, we turn to sample entropy-based analyses, which offer a means of capturing the complexity and dynamical properties of social cohesion over time.

## 3. Main results

In this section, before presenting the results, we recall that sample entropy (SampEn) was used as a measure of the complexity of cohesion dynamics; SampEn is formally defined in electronic supplementary material, eqn. (A 1). The details of how SampEn was computed, including preprocessing steps and entropy calculations, are provided in electronic supplementary material, A.2.

We now begin by statistically assessing whether SampEn varies across the three phases of interest (peace, partial annexation, full-scale invasion). Formally, the null and alternative hypotheses of the Kruskal–Wallis test are given in equation (2.2), and the corresponding test statistic is defined in equation (2.3). Applying this test to SampEn revealed a highly significant effect with $\chi_{\text{cal}}^{2}=106\;\text{and}\;p\text{-value}$ < 10^–5^, indicating that entropy levels are not homogeneous across phases.

**Figure 3.**
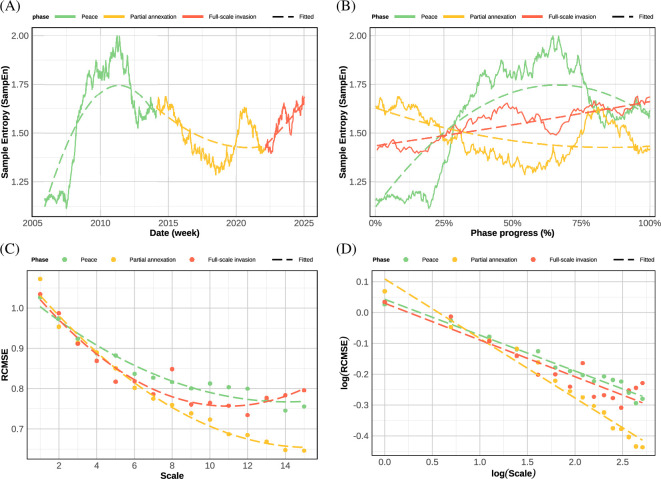
Entropy levels decrease and decay faster during the partial invasion phase (orange) relative to full invasion (red) and peacetime (green). (A) Weekly sample entropy levels from 2004 to 2025. (B) RCMSE across war phases and over time scales τ=15. (C) Interaction plot from the quadratic mixed-effects model of sample entropy dynamics across phases and over standardized phase duration. (D) Log–log plot of the interaction from the general linear model of RCMSE dynamics across phases and over time scales τ=15. Lines represent polynomial (A–C; 2nd order) and linear (D) fits.

We then processed C(t) to remove time trends and seasonal noise. Sample entropy of this processed C(t) series was computed using a rolling window algorithm (N=999; figure 3A). This sample entropy SampEn series was used to characterize small-scale, short-term differences in entropy levels between war phases. In addition, we probed large-scale, long-term dynamics by computing the refined composite multiscale entropy (RCMSE) across each war phase (figure 3B), as defined in [11]. Model selection, parameterization, data transformation as well as entropy algorithm computations are further detailed in electronic supplementary material A.3.

We started with the analysis of social cohesion sample entropy analysis to characterize its short-term behaviour. A mixed-effects model revealed substantial between-phases differences in social cohesion entropy dynamics F(2,176)=29.3, *P* < 0.001, $R^{2}=0.57$ (figure 3C). More specific dynamics were captured by significant linear and polynomial (quadratic) interaction terms between time and war phases ($0.02<P_{s}<0.001$). In terms of shape, peace phase sample entropy displayed concavity, β=−0.12, 95% CI [−0.15,−0.09], P<0.001, while both war phases did not display substantial quadratic behaviour ($P_{s}>0.10$). In terms of trends, social cohesion entropy increased over time under phases of peace, β=0.13, 95% CI [0.09,0.17]*,* P<0.001 and full-scale invasion, β=0.07, 95% CI [0.03,0.10]*,* P<0.001, but decreased over time under partial invasion, β=−0.06, 95% CI [−0.09,−0.02]*,* P<0.001. Overall, it seemed that social cohesion dynamics was the least complex and functional under partial invasion.

Robustness tests indicated that these results remained consistent using a rolling window of 50 or 150 (as opposed to the current 100), including or excluding covariates to account for two major historical events that happened in the span of our data time frame: the ‘Euromaidan’ protests and the COVID-19 pandemic. More specifically, all linear and quadratic interactions remained statistically significant ($P_{s}<0.001$) and displayed similar relative differences. Only one difference appeared when using a window of 50 data points: the observed increase in entropy under full-scale invasion ceased to become significant, with considerably wider parameter confidence intervals and larger error terms.

We then probed the RCMSE of social cohesion to describe its long-term behaviour. Using a log–log transformation to estimate Hurst exponents via model betas [12] (see electronic supplementary material A.3.2), a general linear model revealed once more substantial between-phases differences in cohesion entropy dynamics, F(5,39)=152, P<0.001, R2=0.95; (figure 3D). Social cohesion entropy decreased across scales over all phases ($P_{s}<0.001$). Significant interaction contrasts indicated that the rate of entropy decay was notably more pronounced under partial invasion b=−0.12, 95% CI [−0.14,−0.10]*,* P<0.001, than under peace, b=−0.19, 95% CI [−0.17,−0.21]*,* P<0.001, t(39)=−5.64*,* P<0.001, while no such difference was observed with the rate of decay during full-scale invasion b=−0.12, 95% CI [−0.14,−0.10],P<0.001, t(39)=0.21, P=0.84. This analysis corroborated the singularity of partial invasion entropy signatures, which also displayed the sharpest high-scale decline of all phases. This singularity was confirmed by estimated Hurst exponents for each phase, with H=0.44 pre-2014 and post-2022, while partial invasion yielded H=0.41.

## 4. Discussion

This study reveals that specific signatures of interstate aggression are detectable in the entropy of social cohesion time series. More specifically, the detected differences in entropy dynamics may provide insights into the effects of different types of aggression on social cohesion. As we have seen, phases of low-intensity conflict (partial invasion) are associated with short-term decreases in social cohesion complexity. This observation was replicated with RCMSE, where the partial invasion phase displayed the most pronounced decline in signal complexity over scale, drifting towards blue (versus white) noise. Social cohesion was most anti-persistent under partial invasion. Overall, these results have three main implications: theoretical, methodological and applied.

First, the detectable presence of distinct entropy signatures of war phases constitutes an initial step towards a dynamical systems theory of aggression. Early attempts have conceptualized aggression as the intentional application of ‘energy to an organized whole, increasing its disorder by diminishing its information, its shaping’ [13]. Yet, the second law of thermodynamics implies that the entropy of isolated systems left to spontaneous evolution cannot decrease. Assuming the second law holds for information [14], we propose tentatively that aggression could possibly be understood in thermodynamic terms, like other social phenomena (from teenage births [15] to architectural organization patterns [16]). Under such lenses, aggression could be conceptualized as a kinetic effort to open a target system and impose order (i.e. disturb spontaneous evolution) to its information structure. Although speculative at this stage, our results show the kinetic energy applied by the 2014 Russian partial invasion of Ukraine may have diminished the complexity of its cohesion information structure. Paradoxically, the 2022 full-scale invasion would have achieved the opposite and ‘revitalized’ Ukrainian national cohesion.

Methodologically, this study adds up to prior applications of entropy metrics and specifically multiscale entropy to physiological and behavioural signals (e.g. gait, ECG, EEG, keystrokes) [17]. Here, we show that relevant information about the structure of social cognitive systems (i.e. social cohesion) can be extracted and analysed to provide insights into their underlying phase-states. More specifically, our findings echo prior research showing that the multiscale complexity of ECG time series degrades with age and pathology [18]. Here, exposure to low-intensity warfare can be thought of as triggering a form of societally pathological state in terms of cohesion.

At this point, we must note a few caveats which constrain the inference that can be made from the present study. Importantly, the extent to which our operationalized cohesion index—online search behaviour—reflects real-world cohesion (in a war zone) is limited. Issues such as internet access, language choice (Ukrainian versus Russian), and regional digital divides could potentially affect the internal validity of our measure. Similarly, the population displacements induced by the war are susceptible to having affected the representativeness of the Google users underlying the searches, which could also affect the external validity of our findings.

Within the boundaries of the above-mentioned limitations, the insights provided by our analyses could be applied to the monitoring and prediction of social systemic dynamics. Sample entropy and RCMSE series computed from text data could provide useful insights into the real time ‘health’, or resilience of social cohesion. These indices could be used to study the effect of significant events (i.e. wars, elections, natural disasters) on public resilience. Similarly, they could provide useful data to measure the effect of policies which aim to foster social wellbeing, trust and resilience (e.g. immigrant integration, policing, welfare). To be of applied value, however, the current study should be replicated across a large sample of countries and using different perturbations. Additional types of analyses could be explored, such as RQA and MFDFA [19].

## Data Availability

All study data are included in the article and/or electronic supplementary material [20]. The datasets supporting the findings of this study are available from the Open Science Foundation repository at https://osf.io/nrqvb/?view_only=4136d753c2d643188f52ca2bc9a83c33.
